# *Legitimacy—*not Justice*—and the Case for Judicial Review*

**DOI:** 10.1093/ojls/gqac009

**Published:** 2022-05-24

**Authors:** Tom Hickey

**Keywords:** judicial review, legitimacy, democracy, Judicial Power Project, Rawls, republicanism

## Abstract

Sceptics of judicial review—from Jeremy Waldron to those in the Judicial Power Project—have tended to attribute to their opponents an erroneous prioritisation of ‘justice’ over ‘legitimacy’. They claim that those who make the case for judicial review do so on the grounds that ‘judges know best’, and that judicial review therefore helps promote the overall justness of a state’s social order—rather than on the grounds that it helps enhance the overall legitimacy of a state’s authority. This article interrogates that line of attack. It explores its roots in political theory, particularly the idea that those guilty of it (such as Aileen Kavanagh) follow in John Rawls’s supposed prioritisation of justice over legitimacy. And it turns to republican and later-Rawlsian thinking on these two concepts to see whether it may offer a sound basis upon which the case for judicial review can be made … legitimately.

## 1. Introduction

In his ‘Gray’s Inn Lecture’ delivered at the launch of the Judicial Power Project (JPP), John Finnis hits upon a line apt to stir the troops in the war against judicial despotism. The ‘drift everywhere towards the subjection of legislative to judicial power’, he ventures, can be attributed to the

discourse in law schools and courts [which] increasingly locates its participants in a universe of standards of correct thought and decision, and of the incorrect and unacceptable, which are generated and shared among persons who speak as if they were nowhere in particular.^1^

Finnis does not cite John Rawls in his lecture, but the line evokes the image of the latter’s ‘original position’. Only in that ‘nowhere’—sealed off from the corrupting influences of politics and passion—can we identify what justice really is. Justice is not what ordinary people think it is. Justice is what liberal intellectuals who think like John Rawls think it is. Justice is prisoners’ rights and euthanasia, and blocking the triggering of Article 50.

Finnis’s line thus evokes that sense of condescension that he and others in the JPP feel on behalf of citizens outside of what he dismisses as the ‘academic, NGO, judicial echo chamber’. To paraphrase Jeffrey Goldsworthy in another address to the JPP, how dare the ‘tertiary educated’, *Financial Times*-reading, ‘professional class’—having ‘lost faith in the ability of their fellow citizens to form opinions about public policy in a sufficiently intelligent, well-informed, dispassionate and carefully reasoned manner’—seek to surreptitiously impose their liberal conception of justice on their fellows through their acquiescing in, or support for, this drift towards juristocracy.^2^ This is an ‘insult to a free people’, as Richard Ekins puts it.^3^ Or, in Noel Malcolm’s words, ‘unless we can be sure that infallibly wise judges can solve all problems involving fundamental values in an objectively correct way, we should do well to maintain some residual respect [for ordinary people and thus] for democratic politics’.^4^

The JPP is not especially focused on theoretical questions—as I am in this article. But their thinking here chimes with (and I dare say is informed by) the theoretical arguments proffered by those we might think of as their intellectual first cousins. The leading figures of contemporary political constitutionalist theory, Jeremy Waldron and Richard Bellamy, also emphasise the notions of ‘insult’^5^ and ‘unpleasant condescension’^6^ on precisely these grounds.^7^ But Waldron and Bellamy are explicit in their identification of Rawls as the bogeyman. Waldron opens *Law and Disagreement* (and later, *Political Political Theory*) by pinning on Rawls the failure of contemporary theorists to appropriately account for the fact of disagreement on justice. ‘Since the publication in 1971 of John Rawls’s book *A Theory of Justice*, political philosophers have concentrated their energies on contributing to, rather than pondering the significance of, disagreements about justice.’ Each is aware of rival views, says Waldron, yet focuses only on offering their own take to the world. It is ‘rare to find a philosopher attempting to come to terms with disagreements about justice within the framework of their own political theory’.^8^ Bellamy likewise sees Rawls as having assumed that in a ‘well-ordered’ society every reasonable citizen basically agrees on what justice consists in, and he sees this assumption as lying at the heart of the ‘legal constitutionalism’ to which he is so opposed.^9^

Here, then, is how I read the underlying conceptual impulse shared by these scholars (whom I shall refer to as ‘sceptics’ of judicial power). They recognise and attribute great importance to a distinction between two of the most basic concepts in political theory: *justice* and *legitimacy*. The distinction must be a difficult one to comprehend, because some of the most eminent political theorists have apparently overlooked it. Rawls himself is said to have done so.^10^ Ronald Dworkin is said to have followed Rawls in the error.^11^ And Rawls and Dworkin, along with most contemporary theorists, are said to have ultimately followed Immanuel Kant in conflating justice and legitimacy. A John Simmons, the scholar most associated with the point, laments the loss of the distinction to what he labels ‘the Kantian orientation’ of contemporary political theory.^12^

And here is how I understand the distinction. (I present it in a somewhat simplified form for now.) When Finnis refers to ‘correct standards of thought and decision’—as he does in that line with which I opened the article—and when Malcolm talks about solving ‘problems involving fundamental values in an objectively correct way’, I take them to be referring to standards and solutions that are understood to be correct *as a matter of justice*. That is, if someone were to express a view that prisoners should or should not be entitled to vote, or a view that same sex partners should or should not be entitled to adopt children, they would be expressing a view pertaining to the concept of justice. ‘Justice’ tends to be concerned with the quality of the body of laws that affects people’s prospects in life; it has to do with people’s economic and social relations *vis-à-vis* one another. ‘Legitimacy’, on the other hand, tends to have more to do with the processes through which politics is conducted and laws come about. Legitimacy has to do not so much with people’s relations *vis-à-vis* one another (at least not with their *social and economic* relations in that respect),^13^ but rather with people’s relations with their government or state. Legitimacy is concerned with the right of the state—its political-moral authority—to compel people to comply with laws some of which they will inevitably think unjust. Or it is concerned with what it might take for the state, and for its institutions and laws, to be seen by those subject to them as at least respect- and support-worthy.

Now let me clarify how this distinction bears upon the argument I shall make in this article, and—in outline—how that argument will take shape. The first thing to say is that I heartily agree with the sceptics on the idea that legitimacy—*not justice*—is the pre-eminent conceptual consideration when it comes to institutional questions. I think Bellamy puts it best when he says that in the absence of a process ‘all can acknowledge as offering a means of grounding the truth of moral, legal and political decisions, our reasons for adopting any process will have more to do with its legitimating than its epistemic properties’.^14^ We cannot hope to build an institutional system that gives us answers to rights questions that *all will agree* are true or just. People in free societies have always disagreed about those questions and they always will. Reasonable people in free societies—including reasonable people who are intelligent, well meaning, other-regarding and informed—have always disagreed about those questions and they always will. And we simply must recognise and seriously grapple with that fact, particularly when designing and defending institutions whose purpose is to authoritatively resolve those very disagreements.^15^

Where I begin to disagree with the sceptics is in respect of the role these two concepts—justice and legitimacy—have played in the scholarly arguments made in favour of judicial review. These sceptics, I suggest, impute to their opponents (ie those of us whom I shall refer to as ‘proponents’ of judicial review) the view that justice—*not legitimacy*—is the pre-eminent conceptual consideration in these debates around judicial power. Now I do not think the sceptics are entirely wrong on the point. As I hope to show, it is arguable that some of the leading proponents are not as clear as they might be as to the distinctiveness of the concepts. But if the sceptics are not entirely wrong to impute this priority of justice to their opponents, they are largely wrong. And part of my argument in the article is to show how this is so.

But there is another part of my argument, and it is directed at those proponents of judicial review rather than at the sceptics. It is directed in particular at those—the likes of Jeff King and Aileen Kavanagh, Rosalind Dixon and Frank Michelman, Dimitrios Kyritsis and Mattias Kumm, among several dozens of others—who make their case for judicial review from a broadly Rawlsian and/or neo-republican theoretical base (or who would themselves be broadly supportive of Rawlsian and/or neo-republican ideas on justice—ie those liberal intellectuals whom Finnis would appear to have in mind in that line with which I opened).^16^ My concern in this article is less with the particulars of what these scholars say in defence of judicial review—though I do consider some aspects of those particulars in respect of a small selection (section 5). It is more with the *theoretical grounds of their defence*, particularly as they pertain to these two most basic concepts in political theory. I thus take the republican case first (section 3), exploring whether Richard Bellamy is right to depict Philip Pettit—the leading republican theorist of the age—as having rooted his support for judicial review in an erroneous prioritisation of justice. I then explore whether Waldron is right to depict Rawls—the leading liberal theorist of the age—as having drawn roughly the same institutional conclusion from roughly the same theoretical error (section 4). And my primary aim in those two parts is not so much to show how Waldron and Bellamy are mistaken (much though I think Waldron in particular is) as to clarify and elaborate how legitimacy is in fact understood in their work. It is to show how their thinking on the concept, and on its relationship to justice, evolved over time in their writings—particularly in Rawls’s case. And it is to show how profoundly important this is for contemporary debates on judicial power. My aim in the article, therefore, is to clarify and develop the theoretical grounds of the case for judicial review.

But first I must set the scene (section 2). I must clarify the concepts of justice and legitimacy, and the distinctions between them. I must explain how it is that Rawls and the ‘new Kantians’ came to blur those distinctions. And I must show how the sceptics suppose that their opponents—those who make the case for judicial review—follow in this new Kantian error.

## 2. *Justice—*not Legitimacy*—in the Liberalism of John Rawls*

For present purposes, the main point in AJ Simmons’s seminal work is that the ‘legitimacy of a state with respect to you and the state’s other moral qualities are simply independent variables’.^17^ Your state might score very well in respect of the efficiency or predictability with which it deals with you and your fellow citizens, in its generosity to people in other states, in helping keep some rogue superpower in check or in contributing to the reversal of global warming. These are all good things about your state, and you are right to be pleased about them. But none of them gives it the political-moral authority to force you to comply with its laws—any more than the efficiency, generosity or usefulness of an insurance company gives it the right to force you to give it your business.^18^ And the same is true if your state scores well in respect of the justness of its social policies. Sure, it might facilitate equal access to healthcare, marriage, education and other such goods (euthanasia?) so that it scores well in your eyes in the domain of justice. And that is a good thing (as far as you are concerned) about your state. But it is not what gives it the right to rule over you—if it has such a right at all. It does not mean it scores well in the domain of legitimacy.

I shall ignore what Simmons has to say about what legitimacy itself consists in—in this article I am interested in republican and liberal thinking on that particular question.^19^ But what does interest me about Simmons’s work is what he has to say about the ‘Kantian orientation’ of contemporary political theory—the tendency to treat legitimacy and justice as one and the same. Writing in 1999, Simmons took this orientation to have ‘effectively replaced’ his preferred Lockean position among political theorists, under which these concepts are understood as distinct.^20^ And much like Waldron in *Law and Disagreement* and *Political Political Theory*, he pins it on Rawls, whose work presents ‘if not a simple conflation of questions about justification [justice] and legitimacy, at least a very distinct narrowing of the differences between the argumentative grounds for claims of [justice] and legitimacy’.^21^

Now, there can be no doubt but that Rawls had the concept of justice primarily in mind throughout most of his career. He is famous, after all, for having written a book called *A Theory of Justice*, and on its very first page he describes justice as ‘the first virtue of social institutions’.^22^ He frequently makes corresponding assertions, such as that ‘the fundamental criterion for judging any procedure is the justice of its likely results’.^23^ And at one point in *Theory*—as it happens, it is while he is pondering the justification of judicial review and other such ‘mechanisms of constitutionalism which limit the scope of majority rule’—he gives a sympathetic hearing to Mill’s view that persons with greater intelligence and education might be granted extra voting power.^24^ He does not endorse Mill’s position, and he is careful with his wording throughout the passage.^25^ But he is reassured by his sense that Mill’s justification for it corresponds with his own difference principle. Mill is reasoning ‘from the perspective of those who have the lesser political liberty’, says Rawls, or on the basis of the view that ‘the inequality of right would be accepted by the less favoured in return for the greater protection of their other liberties that results from this restriction’.^26^ And it is here that Rawls lets slip the view that ‘the political liberties are indeed subordinate to the other freedoms’.^27^ The view, we might say, that legitimacy is subordinate to justice.

These aspects of Rawls’s work are understandably seized upon by the likes of Waldron and Bellamy. But they are not quite the focus of Simmons’s critique. Simmons’s main gripe with Rawls—the basis for his designating him a ‘new Kantian’, guilty of conflating justice and legitimacy—is his displacing of a concern for what *actual people* actually accept with one for what *hypothetical people* might be expected or ought reasonably to accept.^28^ And this shift is fundamental to the argument I develop in the article. It means that political power is thought legitimate with respect to a set of persons ‘if it would be reasonable for them to endorse it’.^29^ (Note the tense Rawls employs.) And it means that the emphasis is no longer so much on the question of *how* a social order is imposed on citizens, but rather on *what* social order might be chosen in the original position—on the substantive nature or quality of the social order. The emphasis thus shifts from what looks like a fundamentally legitimacy-oriented question (the ‘how’) to what looks like a fundamentally justice-oriented question (the ‘what’).^30^

This reliance on hypothetical endorsement is especially apparent when Rawls invokes the idea of a ‘just constitution’—which he tends to do whenever questions pertaining to legitimacy arise. He introduces the idea in chapter IV of *Theory*, before returning to it in chapter VI, in a discussion of political obligation.^31^ ‘We normally have a duty to comply with unjust laws,’ says Rawls, ‘in virtue of our duty to support a just constitution.’^32^ He returns to the idea again throughout *Political Liberalism* (*PL*), most notably in Lecture IV, when he first presents us with his definition of ‘the liberal principle of legitimacy’. Now we must pay particular heed to this definition, because it is the subject of a number of interesting tweaks in Rawls’s writing throughout the 1990s (as I consider in section 4). But here, in its original form, in the original edition of *PL* (1993), it reads as follows:

Our exercise of political power is fully proper only when it is exercised in accordance with a constitution the essentials of which all citizens as free and equal *may reasonably be expected* to endorse in the light of principles and ideals acceptable to their common human reason. This is the liberal principle of legitimacy.^33^

We thus have a picture of Rawls’s approach to legitimacy at this point in the development of his thinking.^34^ We see that whether a given law gives rise to a presumptive obligation of compliance depends upon its compatibility with this constitution. We learn that this constitution gets to play this foundational role in virtue of its credentials as ‘just’. And we learn that those credentials are determined with reference to the principles of justice identified in the original position (that is, with reference to Rawls’s theory of justice, ‘justice as fairness’). Once the parties in the original position had identified the two principles of justice, explained Rawls in *Theory*, they would ‘move to a constitutional convention’. And as ‘delegates’ to that convention, with the veil of ignorance only ‘partially lifted’, they are to choose ‘the most effective just constitution, the constitution that satisfies the principles of justice and is best calculated to lead to just and effective legislation’.^35^

Thus, legitimacy is indeed parasitic upon justice in the Rawls of this period and—as per Simmons’s point—is understood in terms of appeal to hypothetical rather than actual choice.^36^ Note, though, what Simmons says about what it is that he finds so objectionable about this new Kantian approach to institutional evaluation:

Appeals to hypothetical choice … have a very different moral basis and force than do appeals to actual choice … Appeals to what ought to be chosen … are perfectly impersonal sorts of moral evaluations. [They …] may be experienced as (possibly paternalistic) groundings for external practical constraints. Appeals to what I have actually chosen … by contrast, seem direct and personal. I am constrained only by how I have in fact lived and chosen. This not only makes the moral constraint seem less external and more obvious…It also makes the constraint more likely to be motivationally efficacious…How we have actually freely lived and chosen, confused and unwise and unreflective though we may have been, has undeniable moral significance …^37^

Now is this not precisely what Finnis was getting at in that line in his Gray’s Inn lecture with which I began? In that instance, he was talking about the ‘discourse in law schools and courts’—that this discourse had its moral roots in a kind of ‘view from nowhere universalism’, and that accordingly it was condescending, or resentment-inducing, for ordinary citizens from outside of that learned discourse community.^38^ (It is resentment-inducing, as Finnis caustically puts it, for those ‘who are unskilled in that learned discourse’s latest tropes and precepts, and who fail to measure themselves against the standards of esteem or disesteem that prevail in a given decade in that community or echo chamber’.)^39^ But Finnis might as easily have been talking about Rawls’s idea of a just constitution, and about the legitimating function that it is thought to serve in a liberal political society. That this constitution—with its provision for judicial review, and its ‘fixing, once and for all, the content of certain political basic rights and liberties’ etc—is one that hypothetical citizens ‘*may reasonably be expected*’^40^ to endorse rather than one that flesh-and-blood citizens *actually* endorse. And that as such it is unlikely to be ‘motivationally efficacious’ (in Simmons’s phrase) in the sense that would be required of any such legitimacy pact.^41^

We see these resonances of Simmons’s objection in the other sceptics’ thinking too—when they rail against the condescension they identify in the case for and institutionalisation of judicial review, and against what they evidently see as the reliance of that case on ideas pertaining to the ‘what’ rather than the ‘how’ (ie on ideas around the substantive nature or quality of the social order—on the institution’s supposed facilitation of ‘just’, ‘enlightened’ or ‘correct’ outcomes—rather than on ideas around how that social order comes about). Recall Goldsworthy’s line about the ‘tertiary educated, professional class’, who are ‘attracted to the judicial enforcement of rights partly because it shifts power to people (judges) who are representative members of their own class, and whose educational attainments [and] intelligence … are thought more likely to produce enlightened decisions’.^42^ Recall also Waldron, who routinely refers to understandings of rights as ‘somehow beyond disagreement’.^43^ So too Bellamy, whose earlier work is littered with references to notions of ‘determinate “right answers” to rights questions’, and ‘virtuous and sagacious judges’ whose views on morality are ‘treated as superior’.^44^ (Bellamy seems to me to rein in the references to the Platonic/‘judges know best’ objection in his post-2007 work.)^45^ And Ekins, who refers to those who understand bills of rights as ‘obvious distillations of moral truth’, and an attendant ‘rhetoric which trades on … the truth about human rights to assert a radical superiority of judicial views about justice’.^46^

## 3. *Legitimacy—*not Justice*—in the Republicanism of Philip Pettit*

I have said that the JPP scholars are not especially focused on theoretical matters—that they instead take their theoretical cue from the political constitutionalists. So it is on Waldron and Bellamy that I must really train my lens. I must look beneath what might in their case be little more than surface rhetoric, and explore what is the real theoretical basis of their attribution of this ‘justice—not legitimacy’ thesis to the case that is made for judicial review.^47^ In Bellamy’s case, he situates his political constitutionalism—and his ‘defence [of] democracy against judicial review’—in Philip Pettit’s republican ideal of freedom as non-domination.^48^ And this puts him at odds with Pettit himself, who tends to speak favourably of judicial review—often referencing it as an example of a non-electoral ‘editorial’ mechanism of the kind he sees as critical institutional supplements to vote-based ‘authorial’ processes.

But my quibble here is not so much with the institutional conclusions Bellamy draws from the ideal of non-domination (much as I would tend to quibble with those conclusions) as it is with the theoretical grounding he ascribes to Pettit in respect of the latter’s support for judicial review. He takes Pettit to conceive of constitutional rights ‘as protecting the basic common interests of all individuals’, and judges as ‘impartial arbitrators of this [rights] framework’ whose role in a republican state is to force power-wielders to stay within the limits that that framework sets (thereby securing citizens from arbitrary government).^49^ And he says that this approach of Pettit’s—which he calls a ‘republican argument for the substantive view of legal constitutionalism’—relies upon ‘being able to come up with an objective account of common interests’. Pettit, says Bellamy, imagines that knowledge of the common good, and of the best means to achieve it, ‘is a “science” composed of objectively valid and vindicated truths, as the laws of physics or mathematical proofs are usually thought to be objective’.^50^ And he

attempts to get round the worry that one might be deferring simply to some individual’s or group’s view of common interests by *imagining they have emerged from a hypothetical democratic process*, similar in kind to the contractarian reasoning employed by Rawls.^51^

Now this is quite significant, because Bellamy is not playing around with provocative phrases in order to secure some broader political goal, nor is he vexed that his vision of the world is out of intellectual favour. He is among the leading constitutional theorists currently writing in English. And here, in one of the most widely cited contemporary books in constitutional theory, he is presenting a carefully considered account of the theoretical grounds of the case for judicial review offered by the leading republican scholar of our time. He is also saying that those foundations are weak. He may not cite AJ Simmons in *Political Constitutionalism*, or engage directly with his work. But he is effectively saying that Pettit falls into the ‘new Kantian’ trap; that Pettit places his preferred conception of justice—a republican variant on justice as fairness—at the foundation of the republican state; and that Pettit supports judicial review on the basis that it helps secure republican justice for the citizenry.

However, I think that Bellamy is quite mistaken. Now I should point out that *Political Constitutionalism* was published in 2007—some years prior to *On the People’s Terms*, in which Pettit elaborates a republican theory of democracy.^52^ I should similarly point out that whenever Bellamy attributes to Pettit this account of how common goods are to be identified and realised, he cites writings of Pettit’s from the 1990s—and thus work that came prior to Pettit’s coming to really focus on the concept of legitimacy.^53^ But Bellamy’s reading is in my view based on a misunderstanding of Pettit’s thinking even as it stood at that time.^54^ And this has only become clearer in the light of the greater emphasis Pettit places on legitimacy in his more recent writing.^55^

Let me explain. Consider my use of the phrase ‘forcing’ just now: that Pettit, on Bellamy’s reading, has judges playing that crucial role of *forcing* government and public officials to stay within constitutional bounds, thus ensuring that they track common goods. Well the ‘forcing’ bit is certainly right. The idea has always been there in Pettit’s work: that the people ‘gain the power to force government’ to govern a certain way;^56^ that they enjoy ‘channels of influence that conjoin to form a river of popular *control* [over government]’;^57^ that ‘government should be forced, in the title of the volume, to operate on the people’s terms’.^58^ But the ‘what’ and the ‘how’ bits are wrong. That is, Bellamy is mistaken in his suggestion that Pettit wants government to be forced to abide by the strictures of pre-political rights.^59^ And he is wrong in ascribing to Pettit the view that judges have this special role in a republican democracy: that they stand above the fray, and do this forcing.

As for what Pettit actually has in mind on these aspects, we must consider the critical *temporal* dimension he emphasises in respect of the identification of common interests, and of the realisation of a shared, popular will in a republican state.^60^ For citizens to share equally in a system of joint control over government, explains Pettit, it must be that they enjoy a degree and form of influence over government that is sufficient to *over time* bring about a law and policy direction that each citizen ‘is ready to accept; that each is disposed to find acceptable’.^61^ (Note the tense that Pettit employs.) Now this may be taken to imply that Pettit has an ideal social order in mind: that he envisages a law and policy direction that would do well by his preferred conception of justice (ie by a republican variation on ‘justice as fairness’). But the republican demand is not for a particular social order in any fixed or substantive sense of the idea. Rather, it is that at any given moment, any citizen—any citizen, that is, who is happy to live on equal terms with their fellows—can step back from this or that aspect of the social order and think that, on the whole, *over the longer haul*, the content of that social order is being driven by underlying norms that they can affirm or at least see as relevant.^62^

Now this, too, bears some attention, because it is easily misunderstood, or its implications overstated. I cannot say that it requires nothing at all in the way of ‘agreement’ or ‘consensus’; it requires what we might think of as a kernel of republican agreement, or what Frank Michelman in a similar context refers to as an ‘irreducible grain or element of liberal substance’.^63^ (I would say ‘republican’ substance. I return to this in section 4.) But it is a very modest kernel indeed, and it involves nothing remotely like the ‘standards of correct thought and decision’ referred to by Finnis, or the ‘consensus on the truth of principles of political morality’ referred to by Bellamy. Nor does it require anything like agreement on ‘the fundamentals of a conception of justice’ of the kind attributed by Waldron to John Rawls.^64^

The idea is that in any democratic society—even in a remotely democratic society—a mode of political exchange is likely to emerge whereby citizens tend to argue for their preferred laws and policies on the basis of reasons they expect others to affirm or at least see as relevant. It may not be the sole mode of political exchange: no doubt some will sometimes doggedly insist upon their preferences in the manner of exhausted toddlers. But where people begin to see themselves as equal citizens rather than as masters and slaves, then there is likely to come a pressure to adopt something like a ‘relevant reasons’ mode. Pettit’s claim then is that insofar as this takes root in the public culture, it generates certain kinds of more specific norms over time, which norms gradually—and for the time being—come to constitute ‘points of reference that are manifestly pertinent or relevant, by everyone’s lights, to issues of public policy’.^65^ And then these norms—these ‘commonly avowable norms’—have a bearing on what kinds of policies are seen as plausible, and what kinds are just off the table. They may not dictate this or that policy outcome, but they make some kinds of outcomes more likely and others seem almost unthinkable. (Thus, the republican approach makes no assertion that this or that law or policy is objectively in the common interest—say, because some inalienable right demands it.) And so it is these shared norms that do the heavy law-and-policy lifting over the longer haul. It is these norms that do that ‘forcing’ of public actors. And they thus put a ‘directive and controlling stamp on what is collectively done in the community’.^66^

I shall get to the matter of institutional implications, and specifically to how this connects to debates on judicial power, later. But for now, the point is that Bellamy is mistaken in assuming that Pettit conceives of common goods in ‘objective’ or ‘pre-political’ terms, and in assuming that he imagines them to have emerged from a hypothetical democratic process. The body of commonly avowable norms emerges from within the social and political world—a world comprising flesh-and-blood citizens—not from behind a veil of ignorance. And as such, we should not be surprised that it may at times include some that reflect ‘questionable beliefs and values’, and that ‘may often be deficient, judged from the point of view of this or that conception of justice’.^67^ Nor should we be surprised that it could ‘never constitute a closed set, fixed once and for all’. Rather, various norms operate *for the time being* in a given society, with innovations inevitable over the course of historical time and ‘likely to be triggered by changes in the dispositions of the existing membership and, of course, by changes of membership that occur at any time and across different times’.^68^

Bellamy is also mistaken to suppose that Pettit puts the justice cart before the legitimacy horse. As should be clear from the preceding paragraph, there is no singular conception of justice lying at the foundation of the republican state. Rather, the citizens who play their part under any given institutional structure ‘will each have their own particular conceptions of justice’, including, we can assume, some with broadly republican and others with broadly Thomist, feminist, Rawlsian, environmentalist or conservative conceptions of justice.^69^ They will inevitably ‘struggle with one another, and perhaps divide quite antagonistically, over particular matters of policy’. And as they each engage in public exchanges, they will each ‘be guided by those conceptions, looking for commonly acceptable considerations by which they can hope to draw others to their side’.^70^ But the prior question in the republican state is one pertaining to legitimacy—*not justice*. It concerns whether such engagement is realised, and such struggles resolved, through institutional mechanisms designed so as to promote equally shared popular control.

## 4. *Legitimacy—*not Justice*—in the Republicanism of John Rawls*

What, then, of Waldron and his gripe with Rawls? Well, that notion of an idealised, fixed or pre-political conception of justice (and the concomitant idea that the justice cart is placed before the legitimacy horse) is a good place to start because the argument Waldron makes in his chapter addressing Rawls in *Law and Disagreement* runs along those very lines. He is ‘reluctant to attribute’ to Rawls the conclusion that there is no such thing as reasonable disagreement on matters of justice in a ‘well-ordered society’,^71^ but he is not reluctant to attribute to Rawls the conclusion that the only disagreement on justice is disagreement at the level of detail, or disagreement in respect of how to apply the principles of justice (in other words, that the Rawls of *PL* still places his own particular theory of justice—justice as fairness—at the foundation of a political society organised around Rawlsian ideas). And this renders Rawls’s work of limited practical value, in Waldron’s view, because here, in the real societies of the real world—whatever about the well-ordered society in John Rawls’s world—‘full-blooded disagreement about justice remains the most striking condition of our politics’.^72^

Now I have already given reasons as to why this could indeed be thought a fair account of Rawls’s approach. These include his suggestion that the political liberties are subordinate to non-political liberties, and his reliance on the notion of a ‘just constitution’ for his analysis of the obligation to obey and for his definition of the liberal principle of legitimacy (see section 2). And there are others reasons supporting Waldron’s view, including the fact that Rawls regularly refers to justice as fairness in the original edition of *Political Liberalism* in ways suggesting that he understood it to have that foundational or antecedent role. He describes it variously as ‘the focal political conception [of justice]’,^73^ ‘the political conception shared by everyone’^74^ and the ‘same political conception … that people affirm [in an] overlapping consensus’.^75^ So it cannot be said that Waldron is ascribing to Rawls something that just is not there.

But I think Waldron is arguably overlooking another reading of the Rawls of that earlier period—and I am strongly of the view that he is failing to recognise quite significant developments in Rawls’s later thinking.^76^ Because Rawls does not always talk in those terms. Even in the original edition of *PL*, he occasionally refers to ‘a range of’ different conceptions of justice, and to justice as fairness as ‘*but one example of* a liberal political conception of justice’.^77^ And this resurfaces in ‘Reply to Habermas’ two years later, before emerging forcefully—and now with real consistency—in his ‘Introduction to the Paperback Edition’ (1996) and in ‘The Idea of Public Reason Revisited’ (1997). Rawls now refers routinely to ‘*a family* of reasonable liberal political conceptions of justice’,^78^ and he elaborates on his point about having used justice as fairness in the original *PL* ‘as an example’ of a political conception of justice.^79^ The book, he says, ‘does recognise that in any actual political society a number of differing liberal political conceptions of justice compete with one another in society’s political debates’.^80^ And in an excited letter to his editor at Columbia University Press in 1998 (proposing a fully revised edition of *PL*, which he never got to write), Rawls says that his ‘thoughts keep changing as time passes’.^81^ ‘Many readers’ of the original edition were ‘misled into thinking’ that the book was about justice as fairness, writes Rawls, when in fact it only had a ‘minor role’ as an example of ‘one political conception of justice among others’.^82^

Now the significance of these shifts in Rawls’s thinking on justice (or these clarifications, as he would insist) can in my view only be fully understood when considered in tandem with corresponding shifts in his thinking on legitimacy across the same writings—and, indeed, in tandem with the greater appreciation he comes to show in respect of the extent and (more to the point) the range of reasonable disagreement in democratic societies. (Recall that Waldron opens *Law and Disagreement* with the claim that political theorists have come to ignore the concept of legitimacy since the publication of *A Theory of Justice*, and to concentrate their energies instead, as Rawls supposedly did, on offering their own theories of justice to the world.) We saw the original version of Rawls’s liberal principle of legitimacy back in section 2—which is from Lecture IV of *PL*. Well that resurfaces with only a slight modification in Lecture VI, and again in almost the same form two years later in ‘Reply to Habermas’ (1995).^83^ But the following year again, in ‘Introduction to Paperback’, we see a notable change. Whereas previously the exercise of political power had been thought legitimate ‘only when it is exercised in accordance with a constitution the essentials of which all citizens may reasonably be expected to endorse’ (ie a ‘just’ constitution, understood as such with reference to what would supposedly have been chosen in the original position), now it is thought legitimate ‘only when we sincerely believe that the reasons we offer for our political action may reasonably be accepted by other citizens as a justification of those actions’.^84^ (Note the tense that Rawls now employs.) And the following year again, in ‘Revisited’ (which Rawls in that excited letter described as ‘by far the best statement I have written’ on the ideas of political liberalism), we see it change once more—and this time it is given a new title to boot. It is no longer ‘the liberal principle of legitimacy’, as it had been on each of the earlier occasions; rather, it is ‘the idea of political legitimacy based on the criterion of reciprocity’. And the main line now reads:

Our exercise of political power is proper only when we sincerely believe that the reasons we would offer for our political actions—were we to state them as government officials—are sufficient, and we also reasonably think that other citizens might also reasonably accept those reasons.^85^

Now I understand Pettit to be among the scholars tending to doubt the conceptual significance of these changes.^86^ But on this I take a different view, because they seem to me to be accompanied by (and to chime with) other shifts in Rawls’s thinking across these same writings.^87^ These include his reining in and qualification of the use of the original position, and his placing of more emphasis on the companion idea of reflective equilibrium.^88^ But the major shift is that towards reciprocity in justification—as traced with particular skill in Silje Langvatn’s work.^89^ ‘By what ideals and principles,’ asks the Rawls of ‘Revisited’, ‘are citizens who share equally in ultimate political power to exercise that power so that each can reasonably justify his or her political decisions to everyone?’ His response is that citizens must be prepared to offer one another fair terms of cooperation ‘according to what *they consider* the most reasonable conception of political justice’, and that when those terms are proposed ‘*those proposing them* must also think it at least reasonable for others to accept them’.^90^

In tandem with that qualification of the original position then, Rawls has come to place actual people and what they actually think at the centre of his stage—displacing hypothetical people and what they ought or might reasonably be expected to think. (There is no sense at this stage of his thinking in which the views of any particular body of the citizenry as to what justice consists in is to be ‘treated as superior’, nor that any sources of political or legal insight are to have the status of ‘obvious distillations of moral truth’. Rather, how actual people actually engage—confused, unwise or unreflective though they will no doubt often be—is what matters, and is given its moral due.) And he has come to orient his thinking around ideas that are fundamentally ‘how-’ rather than ‘what-oriented’. That is, he is concerned fundamentally with *how* citizens justify their political arguments to one another as the social order evolves over time—where previously he had been concerned with *what* those justifications might consist in, or with whether the social order did well by his preferred conception of justice, justice as fairness.^91^ (Thus, the distinction between legitimacy and justice was drawn a little too simply in section 1—as I mentioned then. We can now see that legitimacy does actually have to do with citizens’ relations *vis-à-vis* one another, rather than simply, or exclusively, with citizens’ relations with their government or state.^92^) Indeed, this point is further supported by Rawls’s emphasis now on the fact that the members of the ‘family’ of conceptions of justice in play in a given society ‘are not of course compatible and may be revised as a result of their debates with one another’ or as a result of ‘social changes over generations’ or the emergence of ‘new groups with different political problems’.^93^ Rawls now happily grants that ‘any actual society is more or less unjust—usually gravely so’. And he pitches his notion of a ‘just constitution’ as ‘always something to be worked toward’.^94^

Now I hope that the parallels between this later Rawlsian thinking and those republican ideas elaborated in section 3 are apparent, and how we might therefore see these shifts in Rawls’s thinking as reflecting a pivot to legitimacy on Rawls’s part—indeed, a pivot to republican legitimacy on Rawls’s part.^95^ I hope I have shown that in neither case does ideal justice, or a singular conception of justice, lie at a state’s foundation: not in the case of the Rawlsian state as it is elaborated in the Rawls’s Paperback Edition, nor in the case of the republican state as it is elaborated in Pettit’s *On the People’s Terms*. And I hope I have shown how—under each approach—justice and legitimacy, though conceptually distinct, are not to be thought of as existing on two entirely separate conceptual planets (again pointing to that slight simplification in my articulation of the distinction in section 1, and perhaps to an excessive rigidity in AJ Simmons’s views of the point). That is, that under each approach justice is seen as something that can never be fully realised from the perspective of any one ideal conception of justice yet, at the same time, can always be worked towards—and likely to prevail over time to some degree acceptable from most perspectives (ie so long as the institutional structures do well by the ideal of legitimacy).^96^ Thus, in neither case is justice conflated with legitimacy in that ‘new Kantian’ sense, but neither are they entirely unrelated. (I return to this point momentarily, when I discuss judicial review.)

Before I finally turn to institutional implications then, let me return as promised to Frank Michelman’s point about an ‘irreducible grain or element of liberal substance’ in Rawlsian thought—which I have suggested is also present in republican thought. I take Michelman to be referring here to Rawls’s growing emphasis from *PL* onwards on the implicit acceptance by citizens of a liberal democracy of certain very basic norms of civility. It may leave Rawls open to the charge that all must subscribe to ‘standards of correct thought and decision, and of the incorrect and unacceptable’—on pain of being cast aside as ignorant or unreasonable (or of being left outside the NGO, judicial, academic echo chamber). But that charge would be unfair, in my view. We see what Rawls in fact has in mind when he talks in his later writings about what features a conception of justice would have to have in order to count as ‘political’ for his purposes. Thus, whatever might be its overall vision of justice—whether it has conservative, Thomist, feminist or environmentalist aims, or indeed the aims of justice as fairness—a given conception of justice must conceive of citizens as ‘free and equal’, for instance, and it must conceive of political society ‘as a fair system of social cooperation’.^97^ It must condemn the institutions of ‘slavery and serfdom’, as well as ‘religious persecution’, ‘the hideousness of cruelty and torture’ and ‘the evils of the pleasures of exercising domination’.^98^

The idea, then, is that each citizen would present arguments in public exchanges according to what they consider the most reasonable interpretation of a conception of political justice that conforms at least to those basic norms of civility.^99^ And if this culture takes root in a society, then it is likely to over time bring about a law and policy direction that each citizen is ‘disposed to find acceptable’ (as Pettit might put it). Or it is likely to bring about a social order comprising laws to which citizens will see themselves as having a *prima facie* obligation of compliance (as Rawls might put it). If it does not take root, on the other hand—if the bulk of citizens instead work from their interpretations of conceptions of justice that, for example, reject the conception of citizens as free and equal, or support slavery or serfdom—then a democratic society would just not be viable over the longer term (Rawls and Pettit would suppose). If this puts Rawls and Pettit alongside judges, NGO activists and academics in a liberal echo chamber, I take it that Waldron and Bellamy are in there too, along with Finnis and Ekins and Malcolm.

## 5. *Legitimacy—*not Justice*—and the Case for Judicial Review*

Let me begin this closing section with a summation of what I have argued so far. I have argued that the leading sceptics of judicial review have tended to attribute to their adversaries the view that the case for judicial review rests upon the institution’s supposed epistemic properties—on its supposed facilitation of ‘just’, ‘enlightened’ or ‘correct’ outcomes. I have suggested that a case made on that basis would tend to chime with a broader approach in political theory whereby justice was prioritised over or conflated with legitimacy. I have pointed to how those leading sceptics appear to connect those two things: they attribute Rawls’s and Pettit’s own support for judicial review (such as it is) to the prioritisation of justice they identify in Rawlsian and Pettitean political theory, and they associate the support for judicial review among their adversaries in the field of constitutional theory—the likes of Aileen Kavanagh, Mattias Kumm and Jeff King—with the influence upon those adversaries of Pettitean and particularly Rawlsian thinking. And, of course, I have argued that neither Pettit nor Rawls (in the end) prioritised justice, but rather legitimacy—understood with reference to ideas around reciprocity of justification and equally shared popular control.

What I have not done is pointed to properties of judicial review that might answer well to the demands of legitimacy so understood. For the record, I think it does possess such properties (as I have argued in previous work in respect of Mattias Kumm’s case for judicial review, and Rosalind Dixon’s).^100^ Or rather, I think it possesses properties that have nothing to do with notions of Herculean wisdom on the part of judges, or with the idea that they ‘know best’ or possess superior views on justice, which properties might be thought to contribute—however modestly and unevenly—to a ‘relevant reasons’ mode of democratic exchange, to a ‘non-dominated justificational discourse’ or to a public culture informed by reciprocity of justification and commonly avowable norms.^101^ And I think that insofar as that might be thought plausible, then it is plausible in turn to argue that the presence of judicial review may contribute—however modestly and unevenly—to the overall legitimacy of a democratic state. Its presence may tend on the whole to enhance or in some instances instigate this justificational discourse (albeit that of course it could in some ways forestall or distort such a discourse—recalling Bellamy’s more recent emphasis on concerns such as that judicial review might incorporate a status quo bias, for example). And its presence may as such give a citizen additional reason to be disposed to find the social order acceptable overall, notwithstanding that that social order will comprise laws to which that citizen will object (including some laws which will have been imposed in part on foot of judicial review).

But I am reluctant to spend time in this article elaborating on these properties and on how I think they might be thought to so contribute. One reason for my reluctance is that I want to avoid muddying the waters of my argument. I should reiterate that my primary aim here is to clarify and develop the *theoretical grounds* of the case for judicial review. I have tried to show that those grounds must pertain to the concept of legitimacy—not justice. I have attempted to illuminate republican and later-Rawlsian thinking on that concept: to show how that thinking might best be understood, as distinct from how it has tended to be understood (or misunderstood) by leading sceptics. My hope is that those who make the case for judicial review might be more inclined to draw on these theoretical sources and ideas—and, indeed, to develop them further.

A related reason for my reluctance is that questions around how and why this or that property of judicial review would do well by this or that aspect of such a nebulous ideal as, for example, a ‘relevant reasons’ mode of democratic exchange (or the promotion of a politics based on ‘commonly avowable norms’) are, of course, always going to be highly arguable, as well as dependent upon all kinds of empirical considerations (not to mention highly relevant variables such as those around the nature and scope of the judicial review that might be adopted in a given system). A legitimacy test of this republican or later-Rawlsian kind is often going to be indeterminate on particular institutional questions—but this is not a weakness in the theory.^102^ The theory is concerned fundamentally with structuring our thoughts about how to approach those questions.

For similar reasons, I avoid cluttering my article here with engagement in debates around particular models of judicial review. What I would say on that front is that a legitimacy-based approach is going to lean a bit more towards what are sometimes called ‘dialogical’ or ‘collaborative’ models of judicial review, and almost certainly towards ‘process theory’ or ‘institutional approaches’ to judicial review—and away from the approach Ronald Dworkin would seem to have had in mind (ie suggesting a less compromising approach on the part of judges, and which all but assimilates questions of legal interpretation to questions of morality and justice).^103^ I would also say that it is hard to see how the US model (which for some reason still manages to retain its dominant hold over constitutional theorists’ imaginations—my own included) could be defended in light of the ideas on legitimacy elaborated in this article. The temporal dimension in particular—and that emphasis on flesh-and-blood citizens and what they *actually* think—seems unlikely to tolerate, much less to demand, a constitution of the kind Rawls appears to have in mind when he refers to the need to ‘fix, once and for all, the content of certain political basic rights and liberties’. It is likely to demand instead a constitution that is appropriately responsive to shifts in flesh-and-blood citizens’ conceptions of justice over time.^104^ So the relatively flexible Irish Constitution, for example, would seem much preferable to that of the United States—notwithstanding that both systems allow for ‘strong form’ judicial review.^105^

Instead of exploring particular properties of judicial review myself, what I shall do is lift the bonnet of a selection of cases made by others—whom I venture would tend to broadly share the conception of justice defended by John Rawls or a republican variant—to see whether they seem to be moved in their arguments by their preferred conception of justice, as the sceptics suppose, or whether they might be understood instead to work from legitimacy (or whether they may fall into the new Kantian trap, by conflating the two). Consider first a flavour of the arguments proffered by Aileen Kavanagh in her influential response to Waldron’s *Law and Disagreement*. At one point, she says that a state’s institutions

should be designed so that they are likely to make political decisions in accordance with right reason’ and that the design ‘that is most likely to yield morally right answers, or is likely to yield the most morally right decisions, is most justified.^106^

And she goes on to say that, in designing institutions, ‘we should choose procedures which are most likely to fulfil the condition of good government’; that any procedure ‘will be acceptable only insofar as it is designed to yield morally correct decisions’; and that ‘the justice of the outcomes of political decisions is the fundamental criterion for judging political institutions and it determines what political procedures we choose’.^107^

Now if Richard Ekins, Noel Malcolm or Jeremy Waldron could guide their adversaries’ fingers over their keyboards (not that they would ever wish to do so), they could hardly draft a sequence of phrases to better set up their counterargument. (Does Aileen Kavanagh somehow know how we might fulfil ‘the condition of good government’, they might ask, or is she deferring to the bible of John Rawls on that lofty question?) But if we look more closely at the reasons Kavanagh provides for why she thinks judicial review might help facilitate these ends, quite a different picture begins to emerge. She talks, for example, of how some rights violations occur ‘through no fault of legislators’ but rather because they ‘did not have the protection of a particular right in the forefront of their concerns when enacting a particular piece of legislation’. And she suggests that the presence of a review mechanism ‘which can be activated by the person whose right is allegedly violated [may] be a useful way of bringing violations of rights to light and helping parliament to rectify any shortcoming in legislation if they occur’.^108^ She talks of the distinction between formal and effective participation: that the regular representative processes may facilitate the former but not always the latter—as in the case of citizens ‘with concerns of marginal political importance’, or those who lack the ‘political, financial or organizational resources’ of their fellows.^109^ And that judicial review may afford such citizens opportunity ‘to bring their claims before a constitutional court which considers their individual case in an impartial tribunal in light of constitutional principles’ such that, ‘at the very least, one’s government may have to justify their policy decisions, and defend them publicly in court’.^110^

I make no comment on the persuasiveness of these or other particular claims Kavanagh makes on behalf of judicial review (other than to say that I find them broadly persuasive but by no means decisive). My interest, rather, is in how we might understand the form of the claims she makes, because they seem to me to have nothing to do with any special wisdom or virtue ascribed to judges over legislators, but rather with ideas pertaining to—as she herself puts it—‘general institutional considerations about *the way in which* legislatures make decisions in comparison to judges, the factors which influence their decision, and the ways in which individuals can bring their claims in either forum’.^111^ In other words, her claims in defence of judicial review pertain more to questions around *how* a social order is imposed on citizens and *how* that social order evolves over time—legitimacy-oriented questions—than to questions around *what* that social order might ideally consist in. And if there could be more clarity around the distinctiveness of the two concepts in her elaboration of the ideas, we might also recall that point in section 4 about justice and legitimacy, though being conceptually distinct, not representing entirely separate conceptual planets. That is, we see here in Kavanagh’s thinking the notion that although the ultimate justifications for judicial review may tend to pertain fundamentally to the concept of legitimacy, we may nevertheless have reasons to argue (ie reasons having to do with the ‘how’) that it tends over time to help generate outcomes that, as Pettit might put it, are not going to offend too radically against the more central principles of our own particular conception of justice too (albeit that we could never hope it to only ever generate outcomes that answer specifically or exclusively to that conception of justice).^112^

Now I chose to give Kavanagh particular attention partly because of the influence of her argument and partly because, of all the prominent cases, it seems to me to be pitched in a manner that leaves it particularly exposed to that ‘justice—not legitimacy’ charge. But I think that most other prominent arguments for judicial review proffered by those liberal intellectuals who think like John Rawls are best understood along those same ‘legitimacy—not justice’ lines. As mentioned, I have argued in other work that it is true of the cases made for judicial review by Mattias Kumm and Rosalind Dixon—indeed, I draw parallels there between Kumm’s and Dixon’s ideas and the promotion of commonly avowable norms.^113^ And, as should be clear from earlier comments, it is also true of the case made by Jeff King—who similarly makes claims on the basis of specified aspects of certain features of processes of legal accountability which, he argues, gives those features *prima facie* instrumental value.^114^ King’s emphasis is thus also on institutional considerations about *the way in which* courts make decisions in comparison to legislatures rather than on the virtues of judges themselves.^115^ But we also see references in King’s work to certain vague ends (guarding the rule of law, preventing the abuse of powers, protecting rights, etc) that these institutional considerations are thought to help realise—again suggesting the idea that, although distinct, legitimacy and justice are not entirely separate conceptual planets.^116^

Then there are those prominent cases where the scholars are quite clear and forthright that their emphasis is on legitimacy—not justice. Richard Fallon Jr may be the most obvious example—at one point in his work, when discussing legitimacy and justice, he says that ‘we should recognize [legitimacy] as a trumping ideal in the realm of political morality’.^117^ And in line with this, he is at pains to point out that his case for judicial review, though similarly ‘outcome-based’ (ie initially suggestive of a new Kantian-type approach), in no sense rests on the idea that ‘courts are more likely than legislatures to make correct decisions about how to define vague rights’.^118^ There are plenty of other examples too. Alon Harel and Adam Shinar’s ‘right to a hearing’ based case is also especially attuned to the problems attending epistemic justifications of judicial review—I have drawn parallels between their case and the promotion of commonly avowable norms in that earlier work too.^119^ The same is true of ‘collaboration-based’ defences elaborated by Dimitrios Kyritsis and Larry Sager—each of which is consciously focused upon ‘the structural virtues of the judicial process’.^120^ And it is certainly true of the highly innovative ‘Supreme Court as referee’ based case that Frank Michelman has been developing in very recent times. Indeed, Michelman is very much attuned to these shifts in Rawls’s thinking we have been considering—and especially to that notion of a ‘just constitution’ as a ‘project’, or as ‘always something to be worked toward’.^121^

## 6. Conclusion

I accept the validity of rhetoric in the making of arguments. However, I do not think it is good enough for a serious scholar, making a serious argument, to draw upon a claim that he has ‘heard philosophers say’ that we should ignore legitimacy and go straight to justice when deciding upon what decision-procedures to employ in respect of rights questions (particularly when that scholar is so influential as to be relied upon by scholar-activists keen to make political hay on the back of his reputation).^122^ I have heard constitutional theorists say things tending to suggest an underappreciation of the distinctiveness of the concepts of justice and legitimacy, but I have never heard a constitutional theorist say that we should ignore legitimacy and work instead from justice. (And I think Waldron is simply wrong to attribute to Rawls the view that the only disagreement on justice in a well-ordered society is disagreement at the level of detail.) Nor have I heard any proponents of judicial review say or imply anything so preposterous as that judges are ‘infallibly wise’, that their views on morality should be ‘treated as superior’ or that bills of rights contain ‘obvious distillations of moral truth’.

But in the end, this article is less about the *justice, not legitimacy* charge laid by the sceptics, and why it tends to misfire. It is really about the *legitimacy, not justice* thesis—as I hope the title and section headings attest. The motivating aim has been to encourage those who make the case for judicial review to take more care to situate their arguments in political theory that steers clear of that new Kantian trap, and that places the legitimacy horse before the justice cart.^123^ Because Waldron and Bellamy are right about lots of things, but especially about this: we are engaged in debates on the design of institutions whose purpose is to settle disagreements on justice in a way that can ‘command loyalty even in the face of those disagreements’.^124^

## Footnotes

1 John Finnis, ‘The Gray’s Inn Lecture—Judicial Power: Past, Present and Future’ (Policy Exchange 2015) 59.

2 Jeffrey Goldsworthy, ‘Losing Faith in Democracy: Why Judicial Supremacy Is Rising and What to Do about It’ (Policy Exchange 2015) 13.

3 Richard Ekins, ‘Human Rights and the Separation of Powers’ (2015) 34 UQLJ 217, 221.

4 Noel Malcolm, *Human Rights and Political Wrongs* (Policy Exchange 2017) 21.

5 Jeremy Waldron, *Law and Disagreement* (OUP 1999) 15; Jeremy Waldron, ‘The Core of the Case against Judicial Review’ (2006) 115 Yale LJ 1346, 1406.

6 Waldron, *Law and Disagreement* (n 5) 303.

7 Richard Ekins has suggested to me that ‘most of those who have contributed to the JPP’s work are broadly in the political constitutionalist tradition’, but points out that there are disagreements among them. There are, of course, very significant differences between Waldron and Bellamy on the one hand and the JPP scholars on the other—and Bellamy has, of course, sought to distance his arguments from those of the JPP. See Richard Bellamy, ‘The Limits of Lord Sumption: Limited Legal Constitutionalism and the Political Form of the ECHR’ in NW Barber, Richard Ekins and Paul Yowell (eds), *Lord Sumption and the Limits of the Law* (Hart Publishing 2016) 193–212.

8 Waldron, *Law and Disagreement* (n 5) 1. It is for this reason that Waldron regards ‘John Rawls’s approach to constitutional design’ as ‘so misconceived’ (294). In the preface of *Political Political Theory* (Harvard UP 2016), Waldron expresses a ‘hope that the whole package will encourage young political theorists to understand that there is life beyond Rawls, life beyond the abstract understanding of liberty, justice and egalitarianism—not that those issues are unimportant. But there is life in the old institutional questions still’. Again, it reads almost as if Rawls never considered institutional questions (and almost as if Waldron has barely considered Rawls’s post-1993 work).

9 The difficulty, says Bellamy, is that ‘there are limitations to our ability to identify a true theory of [justice] and so to convince others of its truth’. This makes it ‘implausible to regard judges as basing their decisions on the ‘correct’ view of what democratic justice demands in particular circumstances’. There are ‘no good grounds for believing that judges can succeed where political philosophers from Plato to Rawls have failed’. See Richard Bellamy, *Political Constitutionalism: A Republican Defence of the Constitutionality of Democracy* (CUP 2007) 3, 4.

10 See A John Simmons, ‘Justification and Legitimacy’ (1999) 109 Ethics 739, 757–9.

11 ibid 757.

12 ibid 768.

13 As should become clear in sections 3 and 4, I think that legitimacy is concerned in part with citizens’ relations *vis-à-vis* one another, but more with how they engage with one another when important political questions are at stake or are being discussed. On this idea, see especially the paragraph in section 4 accompanying n 92.

14 Bellamy (n 9) 191.

15 ‘Even when we dispute the decision,’ says Bellamy, ‘we will need to feel that we should accept it all the same’. ibid 191.

16 King describes himself as an ‘adherent’ of the ‘tradition of liberal egalitarian democratic theory which to me [ie to King] reaches the high-water mark in the work of John Rawls, but which shares a family resemblance with … the republicanism of Philip Pettit’. See Jeff King, ‘The Democratic Case for a Written Constitution’ (2019) 72 CLP 1, 3.

17 Simmons (n 10) 752.

18 ibid 752.

19 Whereas Simmons defends a philosophical anarchist thesis.

20 Simmons (n 10) 768, 769.

21 ibid 758. See also Waldron, *Law and Disagreement* (n 5) 1–3; Bellamy (n 9) 20–6 and 100–7.

22 John Rawls, *A Theory of Justice* (Harvard UP 1971) 3. I rely on the original edition of *Theory* throughout this article.

23 ibid 231.

24 ibid 228, 232–3, 231.

25 For instance, he says that his account of Mill’s proposal is ‘solely for purposes of illustration’ and that Mill’s view ‘enables one to see why political equality is *sometimes regarded* as less essential than equal liberty of conscience or liberty of the person’. ibid 233 (emphasis added).

26 ibid 231.

27 ibid 233.

28 Simmons (n 10) 759. It may be that there are strong parallels between Simmons’s arguments and those made by Bernard Williams—who similarly opposed Rawls’s contractualism. See Bernard Williams, *In the Beginning was the Deed: Realism and Moralism in Political Argument* (Princeton UP 2005). However, though I pretend to no expertise on Williams, I think there are differences between his approach to legitimacy and the republican and later-Rawlsian approaches I draw upon in this article (see sections 3 and 4). For a compelling application of Williams’s thinking to the debates on political and legal constitutionalism, see Cormac Mac Amhlaigh, ‘Friend or Foe? Bernard Williams and Political Constitutionalism’ (2021) 27 Res Publica 219.

29 Simmons (n 10) 759.

30 Simmons (n 10) 761: ‘Kantians think of institutional evaluation in terms of what ought to be chosen by people—that is, in terms of the moral quality of institutions, what makes those institutions good (virtuous, just, etc)—not in terms of people’s actual choices.’ Hence, says Simmons, that Lockean distinction between the two forms of evaluation is watered down. Both ‘are now grounded simply in showing that it would be reasonable for a particular set of persons to accept a particular form of political/economic organization’.

31 See Rawls, *Theory* (n 22) 195–201.

32 ibid 354.

33 John Rawls, *Political Liberalism* (expanded edn, Columbia UP 2005) 137 (emphasis added). See a slight modification of this line on 217. See further references to a ‘just constitution’ at, for example, 336, 398. Note that I rely throughout this article on this expanded edition of *PL* from 2005. It contains the original ‘Introduction’ to *PL* from the 1993 edition and all eight original ‘Lectures’. (Hence the excerpt above is indeed from that original 1993 edition of *PL*, i.e. from the Lecture entitled ‘The Idea of an Overlapping Consensus’.) The 2005 edition which I have used in writing this article also contains a new ‘Introduction to the Paperback Edition’; a new ninth ‘Lecture’ (which is ‘Reply to Habermas’ from *The Journal of Philosophy*, originally published in 1995); a short ‘Introduction to “The Idea of Public Reason Revisited”’, which includes a letter Rawls wrote in 1998 to his editor at Columbia University Press on the idea of a revision of *PL*; and finally, ‘The Idea of Public Reason Revisited’ (which was originally published in 1997 in the *University of Chicago Law Review*).

34 On this, see Silje Langvatn, ‘Legitimate but Unjust; Just, but Illegitimate: Rawls on Political Legitimacy’ (2016) 42 Philosophy & Social Criticism 132. I am much indebted to Langvatn’s work in this article. See also Frank Michelman, ‘The Question of Constitutional Fidelity: Rawls on the Reason of Constitutional Courts’ in Silje Langvatn, Mattias Kumm and Wojciech Sadurski (eds), *Public Reason and Courts* (CUP 2020) 90–114.

35 Rawls, *Theory* (n 22) 196–7. ‘In framing a just constitution,’ he explained on 198, ‘I assume that the two principles of justice already chosen define an independent standard of the desired outcome.’

36 This latter idea is made crystal clear from a footnote accompanying the “Our exercise of political power” passage in *PL* (n 33 fn 5) and in several other passages in both *Theory* and *PL.* See Rawls, *PL* (n 33) 22–7, 225, 336, 338. See also his comments in *Theory* (n 22) 196–7.

37 Simmons (n 10) 761–2, 763 (brackets in original).

38 Finnis (n 1) 59.

39 ibid 59.

40 Rawls, *PL* (n 33) 137 (emphasis added).

41 The idea I am getting at here is that a citizen who has lost out, or who is being forced to comply with a law they think unjust, is not likely to accept that situation, and stand down, merely on foot of the fact of the compatibility of that law with a ‘just constitution’ (ie a constitution understood as such with reference to the original position and thus on the basis of appeal to hypothetical choice). It is effectively to say to that citizen: ‘This is legitimate *because it is just*—and as such you should comply’. Such a citizen is likely to respond: ‘Who are you to tell me what is just and unjust?’ For more on these ideas, see an article of mine currently in draft, and provisionally entitled ‘Constitution as Legitimacy Pact’. I should say that the phrase ‘legitimacy pact’ is Michelman’s. See Frank Michelman, ‘Is the Constitution a Contract for Legitimacy?’ (2003) 8 Review of Constitutional Studies 101.

42 See Goldsworthy (n 2) 13, 15.

43 Waldron, *Law and Disagreement* (n 5) 3. In the same vein, Waldron exhorts constitutional theorists to ‘spend less time with theorists of justice, and more with theorists of authority and theorists of democracy’ (12). He also talks of an assumption of the possibility of a societal ‘convergence on a single set of principles which add up to *the truth* about justice, rights and the common good’ (305). (emphasis in original). See also Waldron, ‘The Core of the Case’ (n 5) 1371–2.

44 Bellamy (n 9) 79, 177, 183.

45 Bellamy has come to focus more and more on practical or empirical concerns around judicial review—for instance, that it may tend to incorporate a status quo bias and protect vested interests under the banner of individual rights (as per the likes of JGA Griffiths and Samuel Moyn). I think these concerns are important, and that Bellamy may be right that some leading proponents of judicial review tend to underappreciate them. But I am struck by the extent to which Bellamy continues to focus on the US model specifically. These practical or empirical concerns are especially acute in the US system—for all kinds of contingent reasons (not least the difficulties attending amendment of the US Constitution). So if the main argument of Bellamy’s more recent work on the topic is that the US model of constitutionalism falls short on legitimacy grounds—then I am with him. But that leaves open the question of the legitimacy of judicial review in principle (ie including ‘strong form’ models). In a related point, Bellamy of course now embraces ‘weak form’ judicial review, effectively seeing it as a legitimacy-enhancer, but he seems to me to give insufficient attention, in that context, to non-US versions of strong form review. He also seems to give insufficient attention to the notion that any legitimacy-oriented concerns attending strong form judicial review will attend weak form systems too—albeit in different ways and to different degrees. See n 105 below on this—and the paragraph in the main text accompanying it. See also Tom Hickey, ‘The Republican Core of the Case for Judicial Review: A Rejoinder to Richard Bellamy’ (2019) 17 ICON 329. For examples of Bellamy’s most recent thinking on the topic, see Richard Bellamy, ‘The Republican Core of the Case for Judicial Review: A Reply to Tom Hickey’ (2019) 17 ICON 317; Richard Bellamy, ‘Turtles All the Way Down? Is the Political Constitutionalist Appeal to Disagreement Self-Defeating? A Reply to Cormac Mac Amhlaigh’ (2016) 14 ICON 204.

46 Ekins (n 3) 222, 223, 232. Noel Malcolm similarly refers to proponents of judicial review conceiving of the balancing of rights against political interests being carried out ‘in accordance with criteria so objective that the answers it gives are demonstrably correct’. Malcolm (n 4) 37; see also 13.

47 Bellamy has pointed out to me that what he is attributing to Pettit (and to Rawls) in *Political Constitutionalism* is a reliance on an objective/substantive account of legitimacy—whereas Bellamy himself insists upon a procedural account of legitimacy (that is, he takes Rawls to employ an objective/substantive account of legitimacy to ground his two principles of political justice). I tend to disagree with Bellamy on this point—and I think the disagreement goes beyond mere terminology. My suggestion is that Bellamy tends to attribute to Pettit and to Rawls a prior reliance on the concept of justice (ie *not legitimacy*). Now this charge is easier to lay in respect of Waldron than it is in respect of Bellamy: the former is more explicit in distinguishing between justice and legitimacy, and could almost be said to depict Rawls as the big, bad wolf of a Justice, not Legitimacy movement in contemporary political theory (see n 8 above, and the paragraph in the main text which it accompanies). But I suggest that Bellamy—like many others—tends to read Rawls in the manner set out in section 2 of this article. I suggest that—like many others—he has tended to underappreciate the important shifts in Rawls’s thinking from ‘Reply to Habermas’ onwards (see section 4 of this article). And I suggest that dozens of lines and phrases in *Political Constitutionalism* tend to support my position on the point. See eg Bellamy’s suggestion that Rawls ‘assumed that the only possible choice in the original position would be in favour of his principles of justice and that these provide the necessary basis for our disagreements to play out’ (*PC* 106). See also the phrases set out in n 9 above (on Rawls) and in n 51 below (on Pettit). Note also Bellamy’s suggestion that judges are thought so wise as to ‘base their decisions on the “correct” view of what democratic *justice* demands’ (*PC* 4—emphasis added). And note the phrases in the main text accompanying n 44 above.

48 Bellamy (n 9) 260.

49 ibid 163.

50 ibid 168, quoting Robert Dahl, *Democracy and its Critics* (Yale UP 1989) 65–6.

51 Bellamy (n 9) 163–4 (emphasis added). He takes Pettit to conceive of constitutional rights in objective and pre-political terms, and as having a fixed content (*PC* 147). He also takes Pettit to identify such rights/interests in something like Rawls’s original position. He chides Pettit for his overlooking of legitimacy-type concerns attending his designation, as Bellamy has it, of a ‘constitutional court as the best guardian’ of these common interests. ‘No doubt, hypothetical reasoners who are suitably abstracted from their core commitments might be able to reach such a consensual agreement,’ he says. ‘But the dilemma confronting real politics is that no convergence [on the substance of rights questions] is likely’ (164).

52 Philip Pettit, *On the People’s Terms: A Republican Theory and Model of Democracy* (CUP 2012).

53 He cites Philip Pettit, *Republicanism: A Theory of Freedom and Government* (OUP 1997) ch 6; Philip Pettit, ‘Democracy: Electoral and Contestatory’ in Ian Shapiro and Stephen Macedo (eds), *Designing Democratic Institutions* (New York UP 2000).

54 I should point out that in his chapter in Shapiro and Macedo (n 53), which Bellamy cites in a key passage on Pettit in *PC*, Pettit does say at one point (108) that his way of defining common interests is ‘broadly contractualist in spirit’ and ‘owes much in particular to the interpretation of Rawlsian contractualism developed in the work of TM Scanlon and Brian Barry’. So Bellamy has grounds for the depiction of Pettit that he proffers in *PC*. I would argue, however, that the basis of Pettit’s later thinking on legitimacy is very much present even in that earlier work—including, as it happens, in that chapter in the Shapiro and Macedo volume.

55 In *Just Freedom*, his next book following *On the People’s Terms*, Pettit shifted his phrasing slightly, now speaking of ‘social justice’ and ‘political justice’, rather than of justice and legitimacy. But the thinking remains the same. See Philip Pettit, *Just Freedom: A Moral Compass for a Complex World* (Norton 2014).

56 Pettit, *On the People’s Terms* (n 52) 4. See the same idea in Pettit, *Just Freedom* (n 56) 134.

57 Pettit, *On the People’s Terms* (n 52) 4. (emphasis added).

58 ibid 3.

59 Whether these rights are understood in terms of natural law, with reference to Rawls’s original position or, as Bellamy puts it of Pettit, via the substituting of some other such ‘idealized apolitical process for real politics’. See Bellamy (n 9) 164; see also 147–50.

60 Pettit, *On the People’s Terms* (n 52) 269–75.

61 ibid 170.

62 ibid 252–9.

63 This line of Michelman’s is actually from an exchange he and I have had on the matter. I really liked the line and use it with his permission. The idea behind the phrase surfaces occasionally in Frank Michelman, *Constitutional Essentials: On the Constitutional Theory of Political Liberalism* (forthcoming, Oxford UP). Relatedly, I should point out there is no sense—I stress, *no sense at all*—in which citizens are expected to agree on policy questions in Pettit’s eyes. See Pettit, *On the People’s Terms* (n 52) 278.

64 Waldron, *Law and Disagreement* (n 5) 153, 153–4.

65 Pettit, *On the People’s Terms* (n 52) 255, 256.

66 ibid 266.

67 ibid 264.

68 ibid 257.

69 ibid 279.

70 ibid 279.

71 ‘As far as I can tell,’ says Waldron, ‘Rawls says that the idea of public reason is incompatible with the existence of reasonable disagreement about *the fundamentals* of justice.’ Rawls, says Waldron, is ‘unabashed’ about offering the general principles of his own particular theory of justice—justice as fairness—as ‘a criterion of whether political argument is being conducted in accordance with the idea of public reason’. Waldron, *Law and Disagreement* (n 5) 153 (emphasis in original).

72 Waldron, *Law and Disagreement* (n 5) 163. Waldron thus doubts that we should ‘infer anything for our politics—including our constitutional jurisprudence—from the purely theoretical possibility of a well-ordered society as John Rawls understands it’.

73 Rawls, *PL* (n 33) xix (‘Introduction’).

74 ibid xix (‘Introduction’).

75 ibid 147 (‘The Idea of an Overlapping Consensus’).

76 In a footnote in that chapter in *Law and Disagreement*, Waldron grants that ‘an arguable exception’ to his reading of Rawls on this point is provided in a passage towards the end of Lecture IV of *PL*, where Rawls refers to ‘different liberal conceptions [of justice]’ and to ‘ideals and principles markedly different from those of justice as fairness’. Rawls ‘does not dwell’ on the idea though, according to Waldron, who proceeds to critique him effectively on the basis of his rejecting the possibility of principled disagreement on justice. Waldron, *Law and Disagreement* (n 5) 152 fn 3.

77 Rawls, *PL* (n 33) 226 (‘The Idea of Public Reason) (emphasis added); see also 6 (‘Fundamental Ideas’), 167 (‘The Idea of an Overlapping Consensus).

78 ibid xlviii (‘Introduction to the Paperback Edition’) (emphasis added). Justice as fairness, he says at xlvi, is the conception that ‘I believe to be the most reasonable conception’, but ‘I shouldn’t deny that other conceptions also satisfy the definition of a liberal conception’.

79 ibid xxxvi (‘Introduction to the Paperback Edition’).

80 ibid xlvi (‘Introduction to the Paperback Edition’).

81 ibid 438 (‘Introduction to “The Idea of Public Reason Revisited”’).

82 ibid 439 (‘Introduction to “The Idea of Public Reason Revisited”’).

83 ibid 217 (‘The Idea of Public Reason’), 393 (‘Reply to Habermas’). Richard Fallon, citing ‘Reply to Habermas’, suggests that Rawls ‘at a crucial point … begins to push his inquiry towards identifying minimal, rather than ideal, governmental legitimacy’. He says that Rawls’s new test for legitimacy ‘is not whether a constitution is “perfectly just” but whether it is “sufficiently just” or “just enough in view of the circumstances and social conditions”’. See Richard Fallon Jr, *Law and Legitimacy in the Supreme Court* (Harvard UP 2018) 27–8.

84 Rawls, *PL* (n 33) xliv (‘Introduction to the Paperback Edition’).

85 ibid 446–7 (‘The Idea of Public Reason Revisited’).

86 Pettit does not say much about this in his writing, but he does say at one point in *On the People’s Terms* (n 52) 143 that Rawls ‘remains faithful to that same broadly Kantian approach when he later replaces the test of contractual eligibility under a veil of ignorance with a looser, associated test of civic justifiability: the justifiability of a legal order, on the basis of reasons that *ought to be* publicly acceptable to the citizens living under it’ (emphasis added). But Pettit cites there the original edition of *PL*, and *Justice as Fairness: A Restatement* (Harvard UP 2001)

87 I can say that Frank Michelman seems to at least broadly agree with my reading. In an exchange on this article that I relay with his permission, he says that he finds ‘not much to disagree with’ in my argument. He then comments: ‘If there is a space between us, it may be over the depth of the Rawlsian shift from 1993 *PL* to the paperback and “Revisited”. I may make that out to be a somewhat lesser one than you do.’

88 In fact, there were signs of this even in the original edition of *PL.* See the discussion at Rawls, *PL* (n 33) 226–7 (‘The Idea of Public Reason Revisited’). See also 399–400 (‘Reply to Habermas’).

89 Langvatn (n 34) 132–53. Others who suggest a distinctiveness in the later work of Rawls include Gerald F Gaus and David Reidy. See Gerald F Gaus, ‘The Turn to a Political Liberalism’ in Jon Mandle and David A Reidy (eds), *A Companion to Rawls* (Wiley-Blackwell 2014) 233–50; David A Reidy, ‘Reciprocity and Reasonable Disagreement: From Liberal to Democratic Legitimacy’ (2007) 132 Philosophical Studies 243.

90 Rawls, *PL* (n 33) 446 (‘The Idea of Public Reason Revisited’), li (‘Introduction to the Paperback Edition’) (emphases added).

91 In highly insightful recent writing on this, Frank Michelman suggests that the Rawls of this period now tied political legitimacy ‘to the diligence of citizens in assessing the public reasonableness of their actions’, whereas previously he had tied it to ‘judgments of constitutional-legal compliance’. See Michelman, ‘The Question of Constitutional Fidelity’ (n 34) 90–114, 106.

92 See n 13 and accompanying text. I should also point out that it would be too simple to suggest that the Rawls of this later period is unconcerned with ‘what’ considerations, or that he takes legitimacy to be concerned only with ‘how’ and not at all with ‘what’ questions. He does not think that, and neither, I suggest, does Pettit.

93 Rawls, *PL* (n 33) li (‘Introduction to the Paperback Edition’), 446 (‘The Idea of Public Reason Revisited’).

94 ibid 401 (‘Reply to Habermas’). For a corresponding view on which I have relied, see Michelman, ‘The Question of Constitutional Fidelity’ (n 34) 90–114, 109.

95 Rawls of course says in *PL* that he sees ‘no fundamental opposition’ between his theory and a non-perfectionist, instrumental interpretation of republicanism. Rawls, *PL* (n 33) 205.

96 As for Pettit’s case, this idea comes out in particular towards the latter part of ch 5 of Pettit, *On the People’s Terms* (n 52) (see especially 279). In Rawls’s case, it emerges best in Rawls, *PL* (n 33) ‘Reply to Habermas’ (especially 427–31).

97 Rawls, *PL* (n 33) 376 (‘Reply’).

98 ibid 431 (‘Reply’).

99 See Michelman, ‘The Question of Constitutional Fidelity’ (n 34) 106.

100 See Tom Hickey, ‘The Republican Core of the Case for Judicial Review’ (2019) 17 ICON 288, 302–8.

101 The phrase ‘non-dominated justificational discourse’ is Oliver Gerstenberg’s. As he put it to me in a private exchange I relay with his permission, ‘all of these approaches [ie the later Rawls approach, Michelman’s approach, as well as Pettit’s and Habermas’s] converge in their different ways on an idea of a non-dominated justificational discourse’.

102 Rawls says something similar about his theory of justice. See Rawls, *Theory* (n 22) 201.

103 My thanks to Jeff King for encouraging me to articulate this point. King’s approach to judicial power has always seemed to me to be in line with this republican and later Rawlsian thinking. See Jeff King, ‘Institutional Approaches to Judicial Restraint’ (2008) 28 OJLS 409. King is very much attuned to this distinction between justice and legitimacy. See eg the chapter entitled ‘Democratic Legitimacy’ in Jeff King, *Judging Social Rights* (CUP 2012).

104 And the republican thinking especially seems to call for conceiving of a constitution less as a fount of ideal justice and more as a *repository—*a temporary repository, or a repository *for the time being*—for those ‘commonly avowable norms’. I develop this idea in Tom Hickey, ‘Constitution as Legitimacy Pact’, currently in draft.

105 For a sophisticated yet simple account of judicial power under the Irish Constitution, and of constitutional change in that system, see Oran Doyle, *The Constitution of Ireland: A Contextual Analysis* (Hart Publishing 2018) chs 8–10. For thinking that I think is in line with this particular part of my argument, see King, ‘The Democratic Case’ (n 16) 1. King notably suggests that ‘the constitution is something that should belong to each generation in the important sense of authorship’ (33), and that the ‘baseline or starting position’ for any amendment procedure ‘should be that it is respectful of ongoing equal political status—ie not be fiercely counter-majoritarian’ (32). I should add that Richard Bellamy gets close to endorsing this view in his very recent work. See Bellamy, ‘Bills of Rights’ in Richard Bellamy and Jeff King (eds), *Cambridge Handbook of Constitutional Theory* (forthcoming, Cambridge UP), where he refers approvingly to King’s thinking on the point (while also pointing out some competing concerns), and also to the Irish example.

106 Aileen Kavanagh, ‘Participation and Judicial Review: A Reply to Jeremy Waldron’ [2003] Law and Philosophy 451, 460.

107 ibid 462.

108 ibid 477–8.

109 ibid 480.

110 ibid 484, 483.

111 ibid 466 (emphasis added).

112 See Pettit, *On the People’s Terms* (n 52) 279.

113 Mattias Kumm, ‘Institutionalising Socratic Contestation: The Rationalist Human Rights Paradigm, Legitimate Authority and the Point of Judicial Review’ (2007) 1 European Journal of Legal Studies 153’ Rosalind Dixon, ‘The Core Case for Weak Form Judicial Review’ (2017) 38 Cardozo L Rev 2193. We also see this thinking further developed in Rosalind Dixon, *Responsive Judicial Review: Democracy and Dysfunction in the Modern Age* (forthcoming, Oxford UP).

114 See Jeff King, ‘The Instrumental Value of Legal Accountability’ in Nicholas Bamforth and Peter Leyland, *Accountability in the Contemporary Constitution* (OUP 2013) 143, 146.

115 I should also mention Thomas Poole’s compelling article, which—although it has a more doctrinal focus—has much in common with my argument. See Thomas Poole, ‘Legitimacy, Rights and Judicial Review’ (2005) 25 OJLS 697, especially 718–21.

116 That is, suggesting that legitimacy-tending-to-lead-towards-justice idea. See n 96 and accompanying text.

117 See Fallon (n 83) 34.

118 Richard Fallon Jr, ‘The Core of an Uneasy Case for Judicial Review’ (2008) 121 Harv L Rev 1695, 1699.

119 Alon Harel and Adam Shinar, ‘Between Judicial and Legislative Supremacy: A Cautious Defense of Constrained Judicial Review’ (2012) 10 ICON 950.

120 Lawrence Sager, *Justice in Plainclothes: A Theory of American Constitutional Practice* (Yale UP 2006) 21, 72–6, 146–147, 198–207. See also Dimitrios Kyritsis, ‘Constitutional Law as Legitimacy Enhancer’ in Dimitrios Kyritsis and Stuart Lakin (eds), *The Methodology of Constitutional Theory* (Hart Publishing 2022) 211–31; Dimitrios Kyritsis, *Where Our Protection Lies: Separation of Powers and Constitutional Review* (OUP 2017) 7–18. See also Aileen Kavanagh, *The Collaborative Constitution* (forthcoming).

121 Michelman, ‘The Question of Constitutional Fidelity’ (n 34) 112. See especially pages 105–112.

122 Waldron, ‘The Core of the Case’ (n 5) 1371.

123 As well as the republican and later-Rawlsian sources I consider in this article, I venture that Jurgen Habermas’s discourse theory may be another such source, as well as the deliberative theories of democracy elaborated by, for instance, Amy Gutmann and Dennis Thompson. But I am aware of arguments to the contrary.

124 Waldron, *Law and Disagreement* (n 5) 2.

## Notes

Thanks to Frank Michelman, Davy Lalor, Silje Langvatn, Oliver Gerstenberg, Philip Pettit, Ros Dixon, Richard Bellamy, Jeff King and two anonymous reviewers for their really helpful comments on this article. Thanks also to Aileen Kavanagh, for hosting me at St Edmund Hall, Oxford in the period in which the main arguments began to shape. This one is for Amélie.

